# Axl alleviates DSS-induced colitis by preventing dysbiosis of gut microbiota

**DOI:** 10.1038/s41598-023-32527-2

**Published:** 2023-04-01

**Authors:** Su-Min Yee, Harim Choi, Jeong-Eun Seon, Yu-Jin Ban, Min-Jae Kim, Jae-Eun Seo, Ja Hun Seo, Sehyeon Kim, Seo Hee Moon, Chul-Ho Yun, Hyang Burm Lee, Hyung-Sik Kang

**Affiliations:** 1 School of Biological Sciences and Technology, Chonnam National University, 77 Yongbong-Ro, Buk-Gu, Gwangju, 61186 Republic of Korea; 2 Department of Nursing, Nambu University, 23 Chumdan Jungang-Ro, Gwangsan-Gu, Gwangju, 62271 Republic of Korea; 3 Environmental Microbiology Lab, Department of Agricultural Biological Chemistry, Chonnam National University, 77 Yongbong-Ro, Buk-Gu, Gwangju, 61186 Republic of Korea

**Keywords:** Inflammatory bowel disease, Mucosal immunology, Inflammation

## Abstract

Axl is a tyrosine kinase receptor, a negative regulator for innate immune responses and inflammatory bowel disease (IBD). The gut microbiota regulates intestinal immune homeostasis, but the role of Axl in the pathogenesis of IBD through the regulation of gut microbiota composition remains unresolved. In this study, mice with DSS-induced colitis showed increased Axl expression, which was almost entirely suppressed by depleting the gut microbiota with antibiotics. Axl^−/−^ mice without DSS administration exhibited increased bacterial loads, especially the Proteobacteria abundant in patients with IBD, significantly consistent with DSS-induced colitis mice. Axl^−/−^ mice also had an inflammatory intestinal microenvironment with reduced antimicrobial peptides and overexpression of inflammatory cytokines. The onset of DSS-induced colitis occurred faster with an abnormal expansion of Proteobacteria in Axl^−/−^ mice than in WT mice. These findings suggest that a lack of Axl signaling exacerbates colitis by inducing aberrant compositions of the gut microbiota in conjunction with an inflammatory gut microenvironment. In conclusion, the data demonstrated that Axl signaling could ameliorate the pathogenesis of colitis by preventing dysbiosis of gut microbiota. Therefore, Axl may act as a potential novel biomarker for IBD and can be a potential candidate for the prophylactic or therapeutic target of diverse microbiota dysbiosis-related diseases.

## Introduction

Axl is a receptor tyrosine kinase belonging to the TAM receptor family along with Tyro3 and MER, and is expressed on the cell surface of mesenchymal, epithelial, and hematopoietic lineage cells^1,2^. Growth arrest-specific 6 (Gas6) is one of the ligands of the TAM receptor tyrosine kinase family, and Axl has the highest affinity with Gas6 compared with Tyro3 or MER^3^. Axl plays an essential role in cell migration, proliferation, and survival through the activation of PI3K-AKT-mTOR, MEK-ERK, NF-kB, and JAK/STAT pathways^4^. In addition, Axl suppresses the inflammatory response in various tissues, such as the lung and kidney^5–7^, especially downregulation of innate immune response and inflammatory cytokine expression in the gut^8,9^.

IBD is a chronic inflammatory disease in the colon and small intestine caused by many environmental and genetic factors^10,11^. IBD is classified as Crohn's disease and ulcerative colitis, with common symptoms such as abdominal pain, diarrhea, rectal bleeding, and weight loss. In Crohn's disease, inflammatory lesions occur throughout the gastrointestinal tract, including the esophagus, stomach, small intestine, cecum, colon, rectum, and anus, whereas ulcerative colitis occurs primarily in the colon and rectum^10,12^. Although several studies reported that dietary and genetic factors combined with dysbiosis of the gut microbiota cause IBD, the exact pathogenesis of IBD remains unknown^13,14^.

Axl has been known to be a negative regulator for innate immune responses and IBD^9,15^. Tyro3^−/−^Axl^−/−^Mer^−/−^ triple mutant mice (TAM TKOs) exhibit hyperactivation and expansion of splenic dendritic cells (DCs)^15^. In response to the activation of toll-like receptor 9 (TLR9) or of TLR4, Axl^−/−^, Mer^−/−^, and TAM TKO DCs overproduce Interleukin-6 (IL-6) and Tumor necrosis factor (TNF) compared with wild-type (WT) DCs. TAM receptor activation by treatment with their ligand Gas6 inhibits TLR3-, TLR4- or TLR9-induced production of proinflammatory cytokines, such as type I Interferons (IFNs), IL-6, and TNF, by blocking TLR signaling pathways. Axl expression is upregulated by TLR and type I interferon receptor (IFNAR) signaling through the signal transducer and activator of transcription 1 (STAT1) pathway^15–17^. Moreover, the mRNA expression of suppressor of cytokine signaling 1 (SOCS1) and SOCS3, inhibitors of both TLR and cytokine-receptor signaling, are induced by TAM activation that follows TLR ligation. These previous findings suggest that Axl, one of the TAM receptors, negatively regulates the innate immune response. Concerning the negative regulation of IBD, lack of Axl and MER signaling upregulates the production of proinflammatory mediators, such as nitric oxide synthase 2 (Nos2), IL-6, IL-17α, TNF, and IL-12p35, and reduces negative regulators, such as IL-10, TGF-β, and resistin-like molecule-α (RELM-α), of inflammation by suppressing alternative M2 macrophage in intestinal lamina propria of DSS-induced colitis^18^. In addition, Axl deficiency promotes the migration of γδ T cells by upregulating CCL25 expression, resulting in an increased population of γδ T cells in intestinal intraepithelial lymphocytes (IELs)^19^.

The gut microbiota is a complex community of microorganisms, including bacteria, archaea, fungi, and yeast, living inside an organism's digestive tract, which regulates intestinal immune homeostasis and immune response^20,21^. The gut microbiota mainly consists of the four major phyla of bacteria; Bacteroidetes, Firmicutes, Actinobacteria, and Proteobacteria. The Bacteroidetes and Firmicutes phyla comprise most of the gut bacteria, while the Proteobacteria and Actinobacteria phyla represent a relatively small portion^22,23^. Bacteroidetes help maintain intestinal immune homeostasis by producing short-chain fatty acids. Firmicutes, abundant in diabetes and obesity patients, involve energy resorption, and Actinobacteria plays an essential role in maintaining gut immune responses, such as gut permeability, immune cell development, and cytokine production^23^. The Proteobacteria phylum consists of six classes: α-, β-, γ-, δ-, ε-, and ζ-Proteobacteria and includes many pathogens: *Brucella* in α-; *Bordetella* and *Neisseria* in β-; *Yersinia*, *Salmonella*, and *Escherichia* in γ-; and *Helicobacter* in ε-Proteobacteria^24^. Dysbiosis is an aberrant composition of these four major bacteria phyla in the gut, which is the key factor for regulating the progress of IBD^25^. In particular, the overgrowth of Proteobacteria is one of the characteristics of dysbiosis in IBD patients^26,27^. In a mouse model of colitis, dextran sulfate sodium (DSS) triggers the altered composition of the gut microbiota, especially the abnormal expansion of Proteobacteria similar to IBD patients^28,29^. However, the precise molecular mechanism for the dysbiosis-mediated onset of IBD is not understood^30^.

Antimicrobial peptides (AMPs) critically regulate host-microbiota homeostasis and the innate immune system by inducing bacterial membrane rupture and inhibiting bacterial growth^31^. AMPs are composed of defensins, Regenerating islet-derived 3 (Reg3), and cathelicidin families, which are primarily secreted by paneth cells, enterocytes, dendritic cells, macrophages, and lymphocytes^32^. Defensins are small cysteine-rich cationic proteins, primarily classified as α-defensins and β-defensins in vertebrates^33^. α-Defensins are predominantly present in neutrophils, macrophages, and paneth cells of the small intestine and can kill gram-positive and -negative bacteria, fungi, and some viruses. β-Defensins are produced in neutrophils, macrophages, granulocytes, NK cells, and epithelial cells, which cause membrane depolarization and cell lysis by interacting with the membrane of bacteria^34^. Cathelicidins abundant in the lysosomes of macrophages and granulocytes kill the bacteria by inducing membrane rupture and facilitate wound healing^35^. Reg3 is the family of C-type lectins expressed in the intestine, which play a pivotal role in maintaining the innate immune system and immune homeostasis in the gut^36,37^.

Previous studies have reported that the gut microbiota is one of the causes of IBD^25–27^ and that the TAM receptor family is known as a negative regulator of innate immune responses and IBD^9^. However, the molecular mechanisms by which Axl is involved in the pathogenesis of IBD by regulating gut microbiota remain unclear. This study demonstrated the role of Axl in preventing dysbiosis of gut microbiota and specified the alleviating effects of Axl on the progression of IBD.

## Results

### The expression of Axl was increased in DSS-induced colitis mice

**Figure 1:**
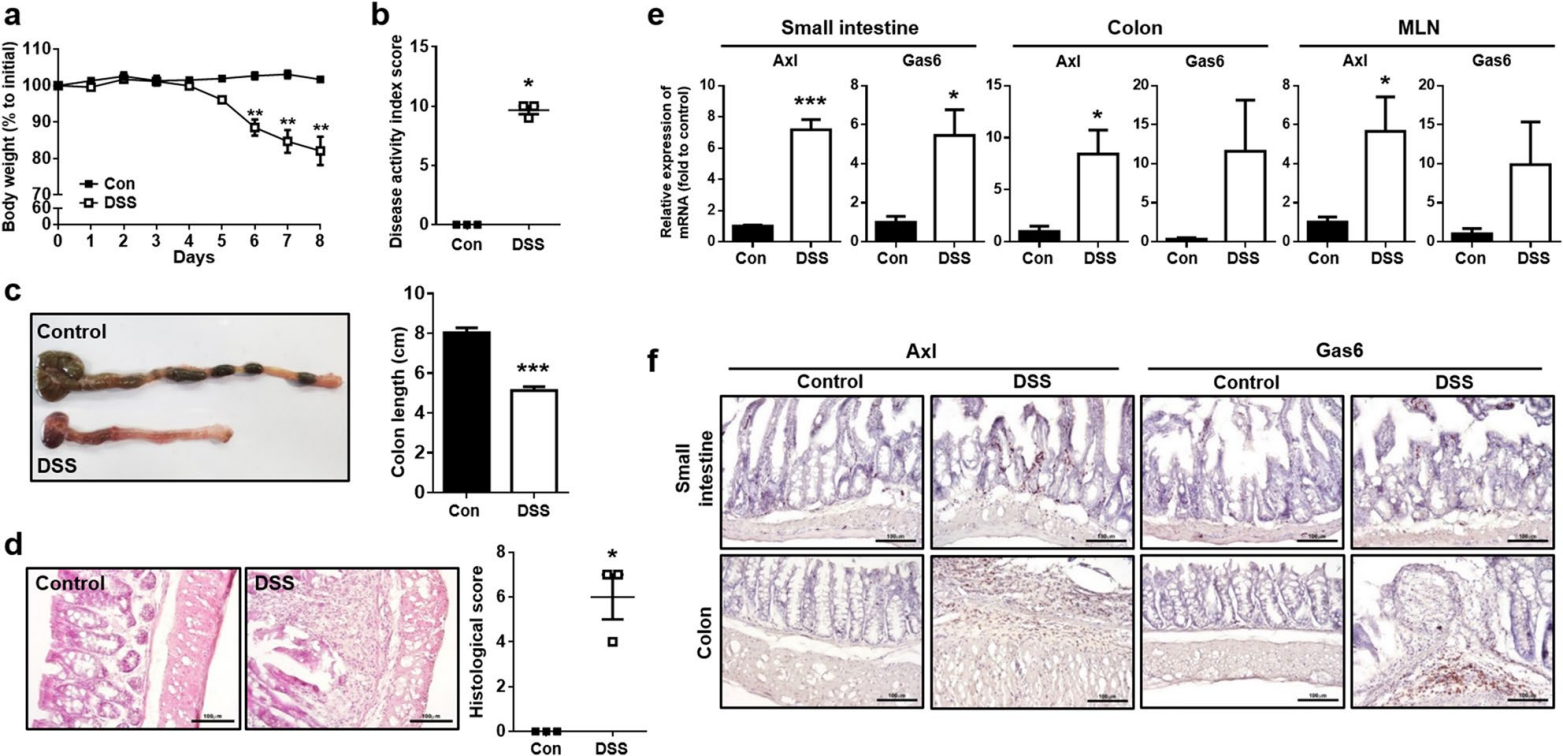
The expression of Axl was upregulated in DSS-induced colitis. (**a**) Changes in body weights were measured during the administration with 2.5% DSS for 8 days. Data are represented as a percentage of body weight relative to initial body weight. (**b**) DAI scores were evaluated based on body weight loss, stool consistency, and bleeding in the stool. (**c**) The macroscopic appearance of the colon (left panel) and the colon length (right panel) were measured on day 8 after DSS administration. (**d**) Representative microscopic images (magnification 200 ×) of colon tissues stained with H&E (left panel). The histological scores were evaluated according to the degrees of epithelial damage, edema, hyperplasia, and immune cell infiltration (right panel). (**e**) mRNA expression of Axl and Gas6 was analyzed by qRT-PCR in the small intestine, colon, and MLN. (**f**) The expression of Axl and Gas6 was evaluated by immunohistochemistry in the colon tissue. The brown-colored areas indicate the expression of Axl or Gas6. Data are shown as the mean ± SEM values. Significance (*p < 0.05, **p < 0.01, ***p < 0.001) was compared with control mice, calculated by the Student's T-test (**a**, **c**, **e**) or Mann–Whitney test (**b**, **d**). Data are representatives of three independent experiments (n = 3 mice per group).

To determine whether Axl is involved in the pathogenesis of colitis, we assessed the expression of Axl in DSS-induced colitis mice. The body weight was decreased in DSS-induced colitis mice compared with control mice (101.7 ± 0.6% in control vs. 82.1 ± 3.2% in DSS-induced colitis mice) (Fig. 1a), and the disease activity index (DAI) score was increased (9.7 ± 0.3 in DSS-induced colitis mice) (Fig. 1b). The colon length was significantly shortened in the DSS-induced colitis mice (8.0 ± 0.2 cm in control vs. 5.1 ± 0.2 cm in DSS-induced colitis mice) (Fig. 1c). The histological score for the degrees of epithelial damage, edema, hyperplasia, and inflammatory cell infiltration was increased in the colon tissue of DSS-induced colitis mice (6.0 ± 0.8 in DSS-induced colitis mice) (Fig. 1d). The mRNA expression of Axl and its ligand, Gas6 was remarkably increased in the small intestine, colon, and mesenteric lymph node in the DSS-induced colitis mice (Fig. 1e). Immunohistochemical analysis revealed that the expression of Axl and Gas6 was upregulated in the colon and small intestine of the DSS-induced colitis mice (Fig. 1f, brown colored areas in the images). These data raise the possibility that Axl might be involved in the pathogenesis of colitis.

### Depletion of the gut microbiota induced the downregulation of Axl expression in the colon of DSS-induced colitis mice

**Figure 2:**
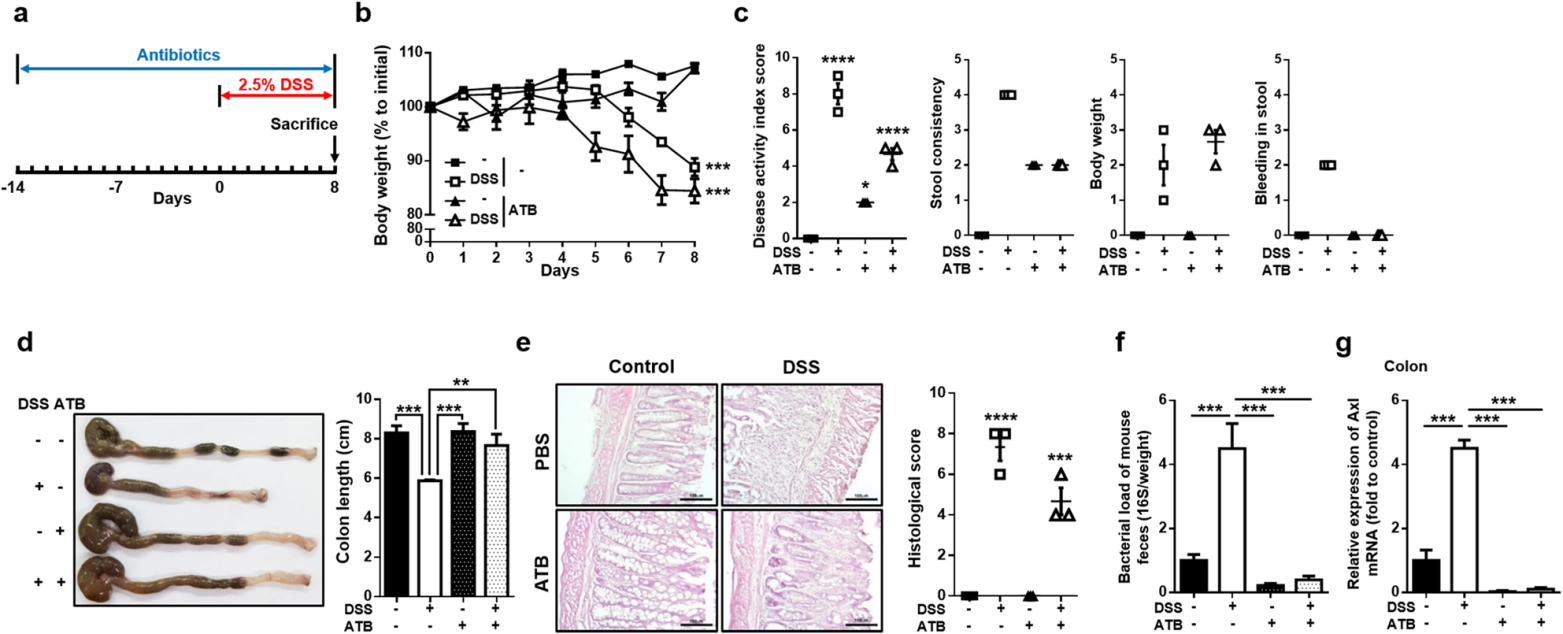
Axl expression was downregulated by the depletion of gut microbiota with antibiotics in DSS-induced colitis. (**a**) Mice were exposed to antibiotics during the entire experiment and administered with 2.5% DSS for 8 days before the sacrifice. (**b**–**e**) Body weights, DAI scores, macroscopic appearance, colon length, microscopic images, and histological scores were evaluated as described in Fig. 1. (**f**) The bacterial load was determined by copy numbers of the 16S rDNA gene per milligram of feces. (**g**) mRNA expression of Axl was analyzed by qRT-PCR in the colon tissues. Data are shown as the mean ± SEM values. Significance (*p < 0.05, **p < 0.01, ***p < 0.001, ****p < 0.0001) was compared with each group and calculated by the one-way ANOVA. Data are representatives of three independent experiments (n = 3 mice per group).

Several studies have reported that the gut microbiota is one of the causes of inflammatory bowel disease (IBD)^13,38^. To examine the effect of gut microbiota depletion on the development of DSS-induced colitis, we exposed mice to antibiotics during the entire experiment and DSS for 8 days before the end of the experiment (Fig. 2a). The body weight loss was higher in DSS-administered mice exposed to antibiotics (ATB-DSS) than in those without antibiotics exposure (Control-DSS) (106.3 ± 0.5% in control; 88.1 ± 1.3% in Control-DSS; 105.7 ± 0.7% in antibiotics-only mice; 80.1 ± 1.8% in ATB-DSS) (Fig. 2b). However, a much higher DAI score was observed in Control-DSS than in ATB-DSS (9.0 ± 0.5 in Control-DSS; 2.0 ± 0 in antibiotics-only mice; 5.0 ± 0.3 in ATB-DSS) (Fig. 2c). The stool consistency score of the antibiotics-exposed mice was increased but lower than Control-DSS (4.0 ± 0 in Control-DSS; 2.0 ± 0 in antibiotics-only mice; 2.0 ± 0.0 in ATB-DSS). Furthermore, there was no bleeding in ATB-DSS, but severe diarrhea and bleeding were observed in control-DSS (2.0 ± 0 in Control-DSS). These inconsistent data between body weight loss and the DAI score may be due to antibiotics' ability to prevent excessive colonic inflammation. Previous studies support the possibility that more body weight loss was observed in ATB-DSS than in Control-DSS, while the DAI score was significantly lower in ATB-DSS than that in Control-DSS^39–42^. The DSS-induced shortening of the colon length was restored to the control mice level by the administration of antibiotics (8.6 ± 0.2 cm in control; 5.9 ± 0.02 cm in Control-DSS; 8.3 ± 0.2 cm in antibiotics-only mice; 7.2 ± 0.3 cm in ATB-DSS) (Fig. 2d), and the histological score was decreased in ATB-DSS compared with Control-DSS (6.0 ± 0.5 in Control-DSS; 4.0 ± 0.5 in ATB-DSS) (Fig. 2e). The administration of antibiotics significantly reduced the increase of DSS-induced bacterial load (Fig. 2f). Next, to determine whether Axl expression can be regulated by the gut microbiota during the progress of colitis, we assessed the expression of Axl in gut microbiota-depleted, DSS-induced colitis mice. Antibiotics administration almost completely inhibited the increased Axl expression in Control-DSS (Fig. 2g). These data suggest that Axl regulated by gut microbiota may be involved in the pathogenesis of colitis.

### A deficiency of Axl signaling induced an abnormal expansion of proteobacteria

**Figure 3:**
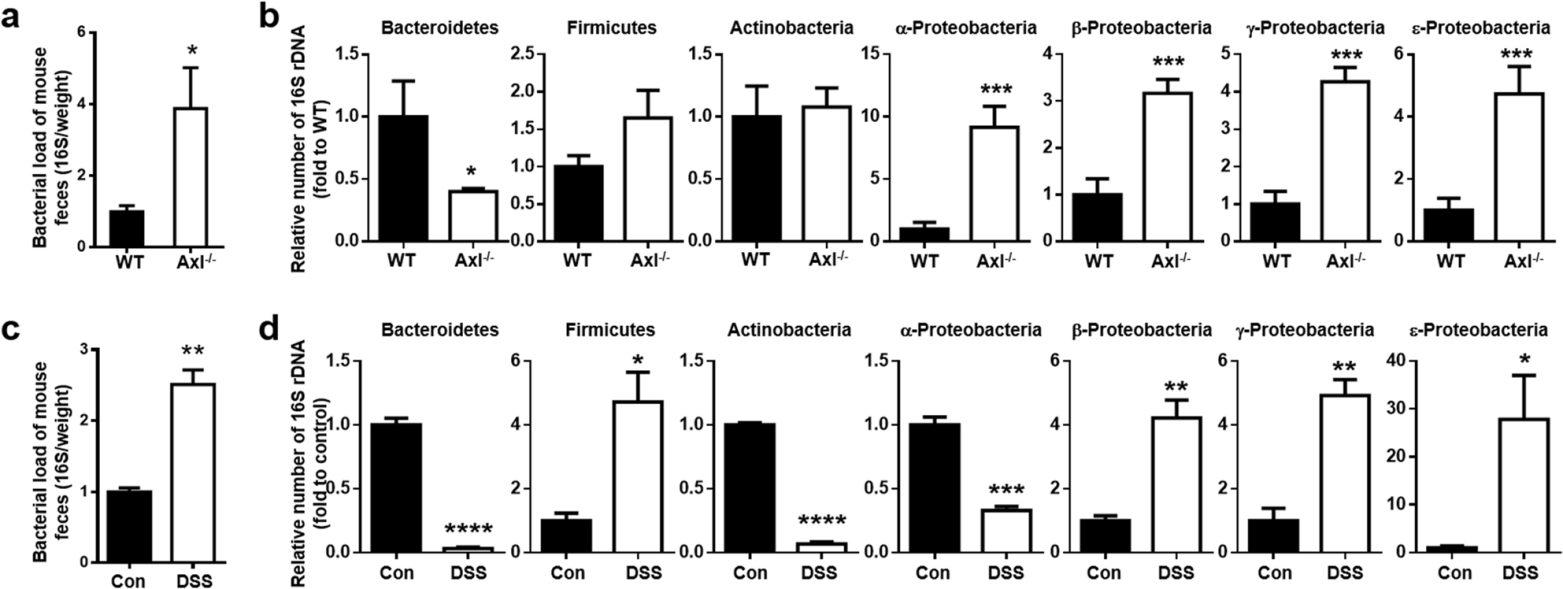
Axl^−/−^ mice showed an abnormal expansion of Proteobacteria. (**a**) Isolation of genomic DNA from feces containing gut microbiota of WT and Axl^**−/−**^ mice and quantification of bacterial 16S rDNA were described in “Materials and methods”. The bacterial load was assessed by copy numbers of the 16S rDNA gene per milligram of feces. (**b**) The relative numbers of bacteria in each phylum were determined with phylum-specific PCR primer pairs. (**c**) The bacterial load in control and DSS-induced colitis mice. (**d**) The relative numbers of bacteria in control and DSS-induced colitis mice. Data are shown as mean ± SEM. Significance (*p < 0.05, **p < 0.01, ***p < 0.001, ****p < 0.0001) was compared with WT or control mice and calculated by the Student's T-test.

To explore the effect of Axl on the composition of the gut microbiota, we analyzed different types of gut microbiota in Axl^−/−^ mice. The bacterial load was increased in the feces of Axl^−/−^ mice compared with WT mice (Fig. 3a). The number of Bacteroidetes was significantly decreased in Axl^−/−^ mice, but all classes of Proteobacteria examined, α-, β-, γ-, ε-Proteobacteria, were markedly increased in Axl^−/−^ mice compared with WT mice (Fig. 3b). No significant differences were observed in the phyla Firmicutes and Actinobacteria between Axl^−/−^ and WT mice. Similar to the data between WT and Axl^−/−^ mice, the bacterial load was elevated by administration of DSS compared with control (Fig. 3c). The numbers of Bacteroidetes, Actinobacteria, and α-Proteobacteria were lower in DSS-induced colitis mice than control mice, in contrast to the increased Firmicutes and β-, γ- and ε-Proteobacteria in the DSS-induced colitis mice (Fig. 3d). These data suggest that Axl^−/−^ mice without DSS administration had an aberrant composition of the gut microbiota, almost consistent with that in DSS-induced colitis mice.

### A deficiency of Axl reduced AMPs and increased inflammatory cytokine expression

**Figure 4:**
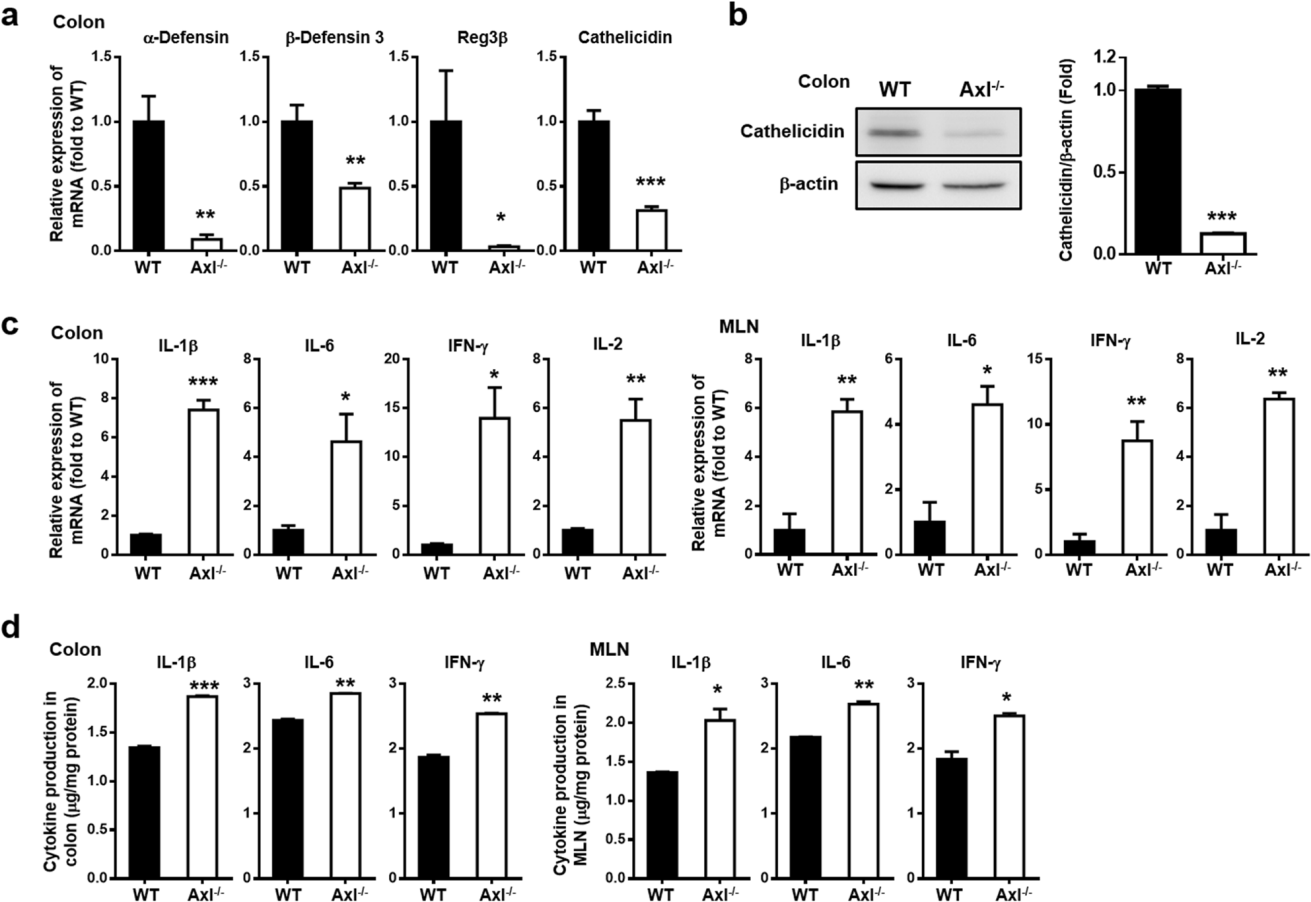
A deficiency of Axl reduced AMPs and increased proinflammatory cytokine expression. (**a**) mRNA expression of AMPs was analyzed by qRT-PCR in the colon of WT and Axl^**−/−**^ mice. (**b**) Protein expression of cathelicidin was analyzed by western blot in the colon. (**c**) mRNA expression of inflammatory cytokines was analyzed by qRT-PCR in the colon and MLN. (**d**) Protein levels of IL-1β, IL-6 and IFN-γ were determined by ELISA in the colon and MLN. Data are shown as mean ± SEM. Significance (*p < 0.05, **p < 0.01, ***p < 0.001) was compared with WT mice and calculated by the Student's T-test.

AMPs have been known to maintain homeostasis between host and microbiota by preventing pathogen infection, modulating immune responses, and interfering with microbial metabolism^31,32^. AMPs, such as α- and β-defensins, also suppress inflammatory cytokine responses through the inhibition of IL-1β secretion and attenuation of IL-1β and IL-6 cytokine signalings^43–45^. To determine whether Axl induces an aberrant composition of the gut microbiota by regulating AMPs and inflammatory cytokines, we analyzed the expression of AMPs and inflammatory cytokines in WT and Axl^−/−^ mice. The mRNA expressions of α-defensin, β-defensin 3, Reg3-β, and cathelicidin were significantly downregulated in the colon of Axl^−/−^ mice compared with WT mice (Fig. 4a). The protein expression level of cathelicidin was reduced in the colon of Axl^−/−^ mice as determined by western blot analysis (Fig. 4b and Supplementary Fig. S1). The mRNA expression of IL-1β, IL-6, IFN-γ, and IL-2 was upregulated in the colon and mesenteric lymph nodes (MLN) of Axl^−/−^ mice (Fig. 4c). IL-1β, IL-6, and IFN-γ protein levels were increased in the colon and MLN of Axl^−/−^ mice as determined by ELISA (Fig. 4d). These results suggest that Axl is capable of restoring the aberrant composition of the gut microbiota by upregulating AMPs and suppressing proinflammatory cytokines.

### Axl alleviated the progression of DSS-induced colitis by preventing dysbiosis of gut microbiota

**Figure 5:**
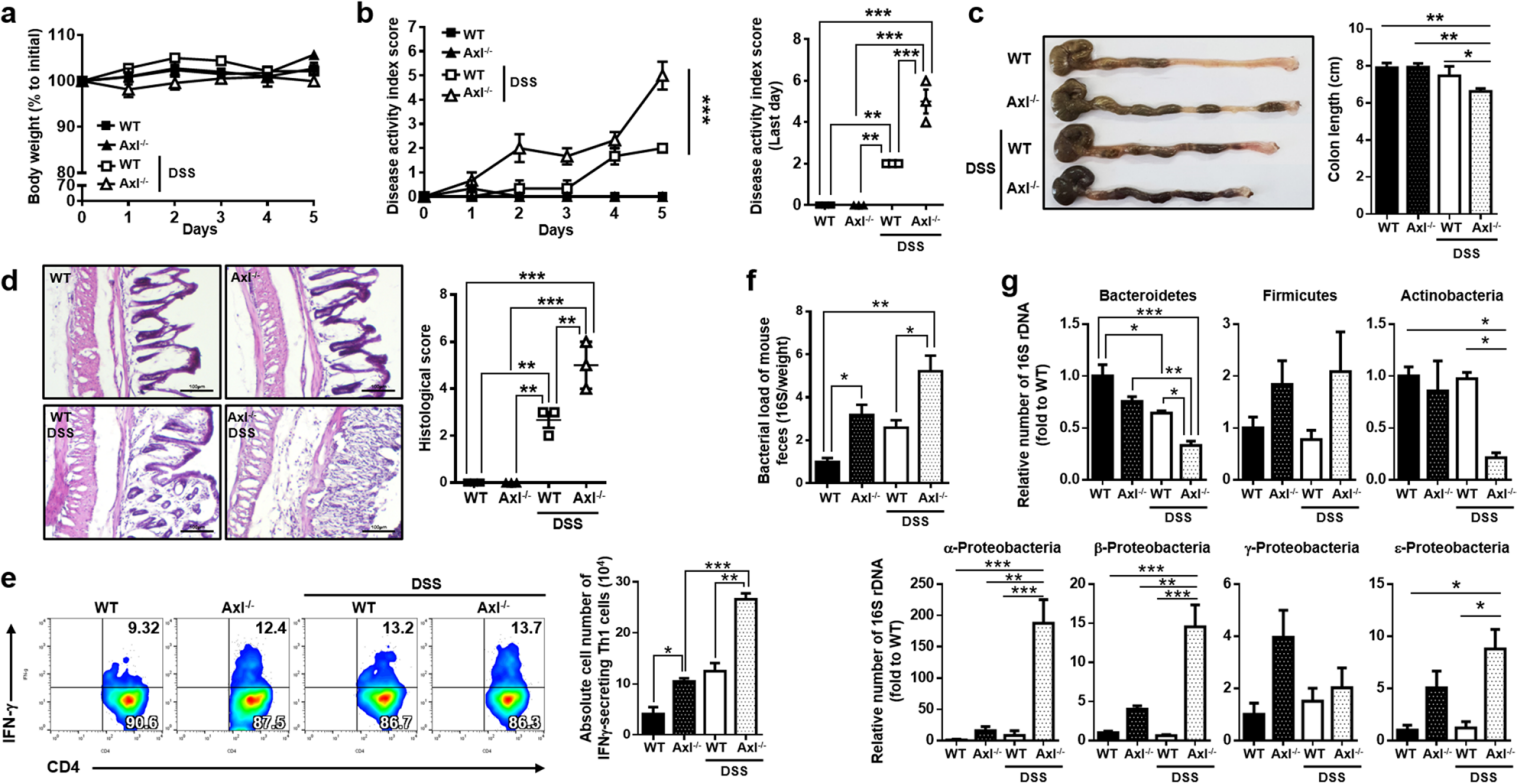
A deficiency of Axl exacerbated DSS-induced colitis by inducing an abnormal expansion of Proteobacteria. (**a**) Body weights and (**b**) DAI scores were measured every day during 2.5% of DSS administration for 5 days. (**c**) The macroscopic appearance of the colon (left panel) and the colon length (right panel) was measured on day 5 after DSS administration. (**d**) Representative microscopic images (magnification 200 ×) of colon tissues stained with H&E (left panel) and the histological scores (right panel) were evaluated as described in “Materials and methods”. (**e**) The populations of IFNγ-secreting activated Th1 cells were determined in the colon using flow cytometry analysis (left panel), and the absolute number of IFNγ-secreting activated Th1 cells was represented as a bar graph (right panel). (**f**) The bacterial load and (**g**) the relative number of bacteria were determined as described in “Materials and methods”. Significance (*p < 0.05, **p < 0.01, ***p < 0.001) was compared with each group and calculated by the one-way ANOVA. Data are representatives of three independent experiments (n = 3 mice per group).

Several studies reported that Proteobacteria is abundant in patients with IBD^46,47^. As shown in Fig. 3, the depletion of Axl increased the bacterial load and Proteobacteria in the gut microbiota. Thus we investigated whether Axl might be capable of alleviating colitis by regulating dysbiosis of gut microbiota. In our preliminary study, the onset of DSS-induced colitis occurred more rapidly in Axl^−/−^ mice than in WT mice. Unlike colitis induction in WT mice, Axl^−/−^ mice exhibited colitis symptoms within 5 days and mortality within 12 days, and thus Axl^−/−^ mice were sacrificed on the fifth day after DSS administration. No significant differences were shown in body weight between Axl^−/−^ (Axl^−/−^ DSS) and WT mice (WT-DSS) administered with DSS (102.7 ± 1.0% in WT control; 105.7 ± 0.4% in Axl^−/−^ control; 102.1 ± 0.7% in WT-DSS; 100.0 ± 0.3% in Axl^−/−^ DSS) (Fig. 5a). As expected, an increase in the DAI score (2.0 ± 0 in WT-DSS; 5.0 ± 0.5 in Axl^−/−^ DSS), histological (2.7 ± 0.3 in WT-DSS; 5.0 ± 0.5 in Axl^−/−^ DSS), and a shortening of the colon length (7.9 ± 0.1 cm in WT control; 8.0 ± 0.1 cm in Axl^−/−^ control; 7.5 ± 0.2 cm in WT-DSS; 6.6 ± 0.1 cm in Axl^−/−^ DSS) were observed in Axl^−/−^ DSS (Fig. 5b–d). The population and the absolute number of IFNγ-secreting activated Th1 cells were increased in the colon of Axl^−/−^ DSS mice (4.1 ± 0.7 × 10^4^ cells in WT control; 10.5 ± 0.3 × 10^4^ cells in Axl^−/−^ control; 12.5 ± 0.8 × 10^4^ cells in WT-DSS; 26.6 ± 0.6 × 10^4^ cells in Axl^−/−^ DSS) (Fig. 5e). These data correlated closely with the data in Fig. 4b, showing upregulated expression of Th1 cytokines, such as IL-1β, IL-2, and IFNγ, as well as previous reports demonstrating that susceptibility to colitis is associated with an increase in Th1 cells^48,49^. The bacterial load of Axl^−/–^ DSS was remarkably increased compared to WT-DSS (Fig. 5f). The numbers of Bacteroidetes and Actinobacteria were decreased in Axl^−/–^ DSS (Fig. 5g), with no significant changes in Firmicutes. Interestingly, α-, β- and ε-Proteobacteria were remarkably higher in Axl^−/–^ DSS than in WT-DSS. These findings imply that the Axl alleviates DSS-induced colitis by suppressing an abnormal expansion of Proteobacteria and plays a vital role in maintaining homeostasis of the host-microbiota in the gut immune system.

### Feces transfer of Axl^−/–^ mice into WT mice administered with DSS aggravated the symptoms of DSS-induced colitis.

**Figure 6:**
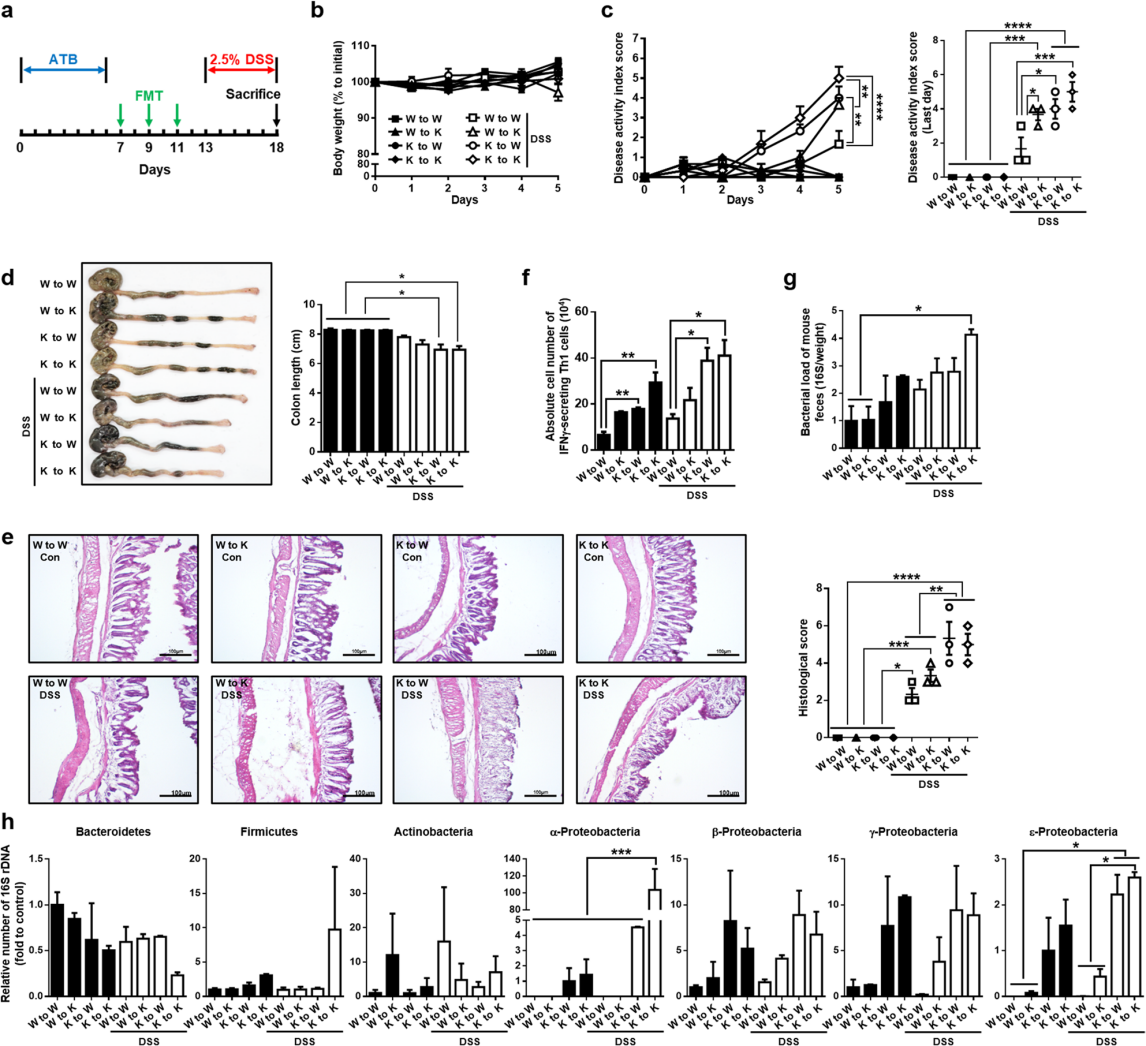
The symptoms of DSS-induced colitis were exacerbated by transfer of feces from Axl^−/−^ mice into WT mice. (**a**) Mice were exposed to antibiotics for 7 days before FMT, and fecal microbiota was transferred 3 times, followed by administration of 2.5% DSS for 5 days before the sacrifice. (**b**) Body weights and (**c**) DAI scores were measured every day during 2.5% of DSS administration for 5 days. (**d**) The macroscopic appearance of the colon (left panel) and the colon length (right panel) was measured on day 5 after DSS administration. (**e**) Representative microscopic images (magnification 200 ×) of colon tissues stained with H&E (left panel) and the histological scores (right panel) were evaluated as described in “Materials and methods”. (**f**) The absolute number of IFNγ-secreting activated Th1 cells was represented as a bar graph. (**g**) The bacterial loads and (**h**) the relative numbers of bacteria were determined as described in “Materials and methods”. Significance (*p < 0.05, **p < 0.01, ***p < 0.001, ****p < 0.0001) was compared with each group and calculated by the one-way ANOVA (n = 3 mice per group).

To validate the role of Axl in the alleviation of colitis by preventing dysbiosis of the gut microbiota, we performed fecal microbiota transfer (FMT). After exposure of mice to antibiotics every other day in a week, FMT was conducted three times a week, and then 2.5% DSS was administered for 5 days (Fig. 6a). The groups of FMT were eight: feces were transferred from WT mice to WT recipients (W to W), WT to Axl^−/–^ mice (W to K), Axl^−/–^ to WT (K to W), Axl^−/–^ to Axl^−/–^ (K to K) with or without DSS administration. There were no significant differences in body weight among the FMT groups (Fig. 6b). Interestingly, in the FMT groups administered with DSS, the symptoms of colitis, including the colon length, DAI, and histological scores, were aggravated when feces were transferred from Axl^−/–^ mice to WT mice (K to W) compared with those of W to W (open symbols and bars) (Fig. 6b–e). The FMT of W to K led to more alleviated symptoms than that of K to K, whereas no significant differences were observed in the symptoms among the FMT groups not administered with DSS (closed symbols and bars). These data, which showed no differences in the symptoms among the FMT groups without DSS, are supported by previous reports that the steady-state gut microbiota cannot induce colitis^50–52^. Furthermore, the gut microbiota is generally innocuous under normal conditions but under adverse conditions, such as DSS-induced colon damage and inflammation, it causes colitis and inflammatory disease. The absolute number of IFNγ-secreting activated Th1 cells was increased in K to W, regardless of DSS administration (Fig. 6f). The DSS-administered K to W exhibited increased bacterial loads and numbers of Proteobacteria, whereas the DSS-administered W to K showed a tendency to reduce them (Fig. 6g,h). These findings suggested that the gut microbiota of Axl^−/–^ mice aggravated DSS-induced colitis by an abnormal expansion of Proteobacteria, and Axl plays a crucial role in suppressing the gut microbiota dysbiosis involved in the pathogenesis of colitis.

## Discussion

Although Axl is known to act as a negative regulator in the progression of IBD, the role of Axl in the alleviation of IBD by regulating the gut microbiota composition remains unraveled. Here, we demonstrated for the first time that Axl signaling might be involved in the pathogenesis of colitis by preventing dysbiosis of the gut microbiota. DSS-induced colitis mice showed an increased Axl expression, downregulated by depleting the gut microbiota with antibiotics. Axl^−/–^ mice without DSS administration exhibited an increased bacterial load, especially in the Proteobacteria abundant in patients with IBD, almost consistent with that in DSS-induced colitis mice. Moreover, Axl^−/–^ mice had reduced AMPs and overexpressed inflammatory cytokines, thereby exacerbating clinical symptoms of colitis. These findings imply that a deficiency of Axl signaling resulted in an aberrant composition of the gut microbiota and an inflammatory gut microenvironment, which exacerbated the colitis. Therefore, Axl signaling may be essential for alleviating DSS-induced colitis by restoring gut microbiota dysbiosis, further raising the possibility that Axl plays a critical role in maintaining the host-gut microbiota homeostasis as a potentially novel biomarker for IBD.

An imbalanced composition of the gut microbiota is known to be one of the causes of IBD^13,38^. In the current study, as shown in WT mice administered with DSS, an imbalanced microbiota composition was found in the gut of Axl^−/–^ mice even without DSS, but no clinical symptoms of colitis, suggesting no causal relationship between the imbalanced composition and colitis symptoms. The lack of a causal relationship may be due to the possibility that steady-state gut microbiota in Axl^−/–^ mice acting as pathobionts that do not induce colitis. The pathobionts are gut microbiota generally innocuous with normal conditions but cause disease under adverse conditions, such as the perturbation of host immune homeostasis, genetic defects, antibiotics administration, or inflammation^50,53–55^. However, DSS-induced colon damage and inflammation can revert pathobionts to pathogenic phenotypes, supported by previous studies that showed DSS triggers the pathogenesis of colitis in the presence of several pathobionts^50–52^.

In this study, we provide the first demonstration that Axl expression was remarkably increased in DSS-induced colitis mice, which led to suppression of the excessive inflammation induced by the colitis consistent with previous studies that Axl signaling inhibits the inflammatory response in various tissues, such as the lung, kidney, and gut^5–7,9^. In addition, Axl^−/−^ mice showed an increased bacterial load compared with WT mice, and antibiotics-mediated depletion of the gut microbiota inhibited the colitis-induced upregulation of Axl, which was corroborated by previous findings showing that the toll-like receptor (TLR) signaling regulates the Axl expression^9,56^. Therefore, our findings imply that Axl and gut microbiota may reciprocally regulate during the progress of DSS-induced colitis.

AMPs maintain host-microbiota homeostasis^31,32^ and regulate the proinflammatory cytokine responses that play a critical role in the progression of IBD as well as the regulation of gut microbiota composition^57–60^. A decrease of α-defensin causes the expansion of Proteobacteria, eventually inducing dysbiosis of the gut microbiota^61^. Depleting cathelicidin reduces the colon mucin layer, causing dysbiosis of the gut microbiota and making the host susceptible to DSS-induced colitis^32,62^. In this study, we provide the first evidence that Axl deficiency caused a dysbiosis of the gut microbiota, especially an abnormal expansion of Proteobacteria, by reducing AMPs expression, as well as excessive expression of proinflammatory cytokines. Previous studies showing that α-defensins and cathelicidins suppress the expression of IL-1β and IL-6 by blocking LPS- or lipoteichoic acid-induced TLR activation^43,63^ support our findings, suggesting that AMPs reduced by Axl deficiency might lead to excessive proinflammatory cytokines.

Proteobacteria is a major bacterial phylum associated with IBD in the gut microbiota^22^, and many pathogens belong to Proteobacteria, such as *Brucella*, *Bordetella*, *Neisseria*, *Yersinia*, *Salmonella*, *Escherichia*, and *Helicobacter*. Several studies have reported that an abnormal expansion of Proteobacteria is one of the characteristics of dysbiosis^26,27^, and the dysregulated host-microbiota interactions cause gut microbiota-related diseases, including IBD^13,25,64^. In this study, the increased Proteobacteria and decreased Bacteroidetes compositions in non-colitis Axl^−/–^ mice were consistent with those in DSS-induced colitis WT mice but dissimilar in Firmicutes and Actinobacteria compositions. This inconsistency may be due to the results obtained from Axl^−/–^ mice not administered with DSS. To compare the compositions of the gut microbiota between WT and Axl^−/–^ mice after DSS administration, we sacrificed WT (WT-D8) mice on day 8 and Axl^−/–^ (Axl^−/–^ D5) mice on day 5 because the clinical symptoms of DSS-induced colitis occurred within 5 days in Axl^−/–^ mice compared with 8 days in WT mice. The microbiota compositions were more similar when comparing Axl^−/–^ D5 mice with WT-D8 mice than when comparing non-colitis Axl^−/–^ mice with WT-D8 mice; Axl^−/–^ D5 and WT-D8 mice showed an increased number of Proteobacteria but a decreased number of Bacteroidetes and Actinobacteria. In addition, WT-D8 mice showed aberrant gut microbiota composition and severe symptoms, whereas WT (WT-D5) mice administered with DSS for 5 days exhibited no significant changes, which might be due to the differences in the severity of colitis in response to the duration of DSS administration. These findings suggest that Axl deficiency makes the gut vulnerability to the onset of colitis by causing dysbiosis of intestinal microbiota.

Although Axl has been known to act as a negative regulator in the pathogenesis of IBD^18^, to the best of our knowledge, the effect of Axl on changes in the composition of the gut microbiota has not been investigated to date. Previous studies have demonstrated that IBD patients show a reduced abundance of the genera *Bacteroides*^65,66^, *Alloprevotella*^67^, *Odoribacter*^68^, *Bifidobacterium*, *Eggerthella*^69^, and *Olsenella* but an increased in *Escherichia*, *Shigella*, and *Actinobacillus*^70^. In addition, the species such as *B. vulgatus*, *B. caccae*, *B. bifidum*, and *B. longum*^68^ are decreased in patients with IBD, whereas the species *S. enterica*^71^, *E. coli*^67^, *A. segnis*, *H. parainfluenzae*, and *E. corrodens*^68^ are enhanced. Among the above gut microbiota, *Bacteroides*, *Alloprevotella*, and *Odoribacter* at the genus level, and *B. vulgatus* and *B. caccae* at the species level belong to the phylum Bacteroidetes^68,69^. The Actinobacteria phylum includes the genera *Bifidobacterium*, *Eggerthella*, and *Olsenella* and the species *B. bifidum* and *B. longum*^68,69^. The Proteobacteria phylum consists of *Escherichia*, *Shigella*, and *Actinobacillus* at the genus level and *S. enterica*, *E. coli*, *A. segnis*, *H. parainfluenzae*, and *E. corrodens* at the species level^68^. In the present study, we found that the numbers of the phyla Bacteroidetes and Actinobacteria were reduced, whereas the phylum Proteobacteria was markedly increased in Axl^−/–^ colitis mice induced by DSS. Therefore, based on the clinical observations of patients with IBD, it is likely that the above-described reduced or increased gut microbiota at the genus and species level might correspond to changes in their compositions at the phyla level in Axl^−/–^ colitis mice induced by DSS.

This study provides the first evidence that Axl may play an essential role in alleviating DSS-induced colitis and maintaining gut homeostasis by preventing gut microbiota dysbiosis. Furthermore, Axl and gut microbiota reciprocally regulate during the progression of DSS-induced colitis, creating a more favorable microenvironment for beneficial gut microbiota by upregulating AMPs and suppressing proinflammatory cytokines. Therefore, Axl may act as a potential novel biomarker for diverse microbiota dysbiosis-related diseases and can be one of the candidates for developing prophylactic or therapeutic targets.

## Materials and methods

### Animals

8-week-old female C57BL6/J mice were purchased from Damul Science (Daejeon, South Korea). Axl^−/–^ mice were kindly donated by Dr.Greg Lemke from Salk Institute, San Diego, CA. The mice were maintained under a 12 h light/dark cycle at 22 ± 1 °C. The animal experiments were conducted in accordance with the guidelines of the Institutional Animal Care Committee of Chonnam National University. All animal experiment protocols were reviewed and approved by the Chonnam National University Institutional Animal Care and Use Committee (CNU IACUC-YB-2021-150), and are in compliance with ARRIVE guidelines.

### DSS-induced colitis mice and antibiotics administration

For induction of colitis, mice were administered with 2.5% (w/v) of dextran sulfate sodium (DSS, MW 36–50 kDa; MP Biomedical, Solon, OH) solutions ad libitum for 5 days or 8 days. The antibiotics cocktail (ATB) for the depletion of gut microbiota was made of 1 g/l ampicillin, gentamycin, neomycin, vancomycin, and metronidazole (Merck, Rahway, NJ), respectively. The ATB was orally administered three times a week during the entire experiment at 200 µl per mouse, and phosphate-buffered saline (PBS) was used as a control.

### Clinical assessment of DSS-induced colitis

Clinical assessment of the severity of DSS-induced colitis was determined as described previously^72^. Briefly, loss of body weight, stool consistency, and bleeding in stool were recorded daily, and the disease activity index score was calculated based on these records.

### Histological analysis and scoring

The colon tissues were fixed with 4% paraformaldehyde, embedded in an optimal cutting temperature (OCT) compound, and stained with hematoxylin and eosin (H&E). Briefly, crypt loss, edema, hyperplasia, and goblet cells in the epithelium were analyzed, and immune cell infiltration into the tissues was observed and scored. The histological scores were determined as described previously^72^.

### Isolation of primary cells from the small intestine, colon, and mesenteric lymph nodes

Tissues from the small intestine and colon were isolated, washed twice with ice-cold PBS, and chopped to 0.5 ~ 1 cm in length. After shaking incubation for 30 min at 37 °C in 10 ml, RPMI-1640 media (GIBCO, Big Cabin, OK) supplemented with 2% fetal bovine serum (FBS, GIBCO), 0.5 mg/ml type IV collagenase (GIBCO), and 20 µg/ml DNase I (Merck), the tissues were filtered with a 100-mesh (150 µm) and centrifuged at 500×*g* for 10 min at 4 °C. To isolate primary cells from mesenteric lymph nodes (MLN), MLN tissue was crushed with a syringe plunger and filtered with a 45 µm nylon strainer, followed by centrifugation at 500×*g*, for 3 min, at 4 °C. The supernatant was discarded, and the cell pellet was resuspended with fresh RPMI-1640 media.

### Quantitative real-time reverse transcription PCR

Total RNA was isolated from the small intestine and colon tissues using TRI reagent (MRC, Cincinnati, OH) according to the manufacturer's instructions. After cDNA synthesis using M-MLV reverse transcriptase (Promega, Madison, WI), the cDNA was added to AccuPower® 2X GreenStar™ qPCR Master Mix (Bioneer) and 10 pmol of primers for each gene. Quantitative real-time PCR (qRT-PCR) was performed under the following conditions: 95 °C for 15 s, 55 °C for 15 s, and 72 °C for 15 s. The sequences of the primers used in qRT-PCR are as follows: GAPDH: Forward, 5′-CAT CAC TGC CAC CCA GAA GAC TG-3′ and Reverse, 5′-ATG CCA GTG AGC TTC CCG TTC AG-3′; Axl: Forward, 5′-GGA GGA GCC TGA GGA CAA AGC-3′ and Reverse, 5′-TAC AGC ATC TTG AAG CCA GAG TAG G-3′; Gas6: Forward, 5′-AAC TCC CCA GGG AGC TAC A-3′ and Reverse, 5′-GCA CGG CAA GAT GTC CTC-3′; IL-1β: Forward, 5′-ACC TGT GTC TTT CCC GTG G-3′ and Reverse, 5′-TCA TCT CGG AGC CTG TAG TG-3′; IL-6: Forward, 5′-AGT TGT GCA ATG GCA ATT CTG A-3′ and Reverse, 5′-AGG ACT CTG GCT TTG TCT TTC T-3′; IFN-γ: Forward, 5′-TGG CAT AGA TGT GGA AGA AAA GAG-3′ and Reverse, 5′-TGC AGG ATT TTC ATG TCA CCA T-3′; IL-2: Forward, 5′-CCT GAG CAG GAT GGA GAA TTA CA-3′ and Reverse, 5′-TCC AGA ACA TGC CGC AGA G-3′; Global α defensin: Forward, 5′-GGT GAT CAT CAG ACC CCA GCA TCA GT-3′ and Reverse, 5′-AAG AGA CTA AAA CTG AGG AGC AGC-3′; β-defensin 3: Forward, 5′-GCT AGG GAG CAC TTG TTT GC-3′ and Reverse, 5′-TTG TTT GAG GAA AGG AGG CA-3′; Reg3β: Forward, 5′-CTG CCT TAG ACC GTG CTT TC-3′ and Reverse, 5′-ATA GGG CAA CTT CAC CTC AC-3′; Cathelicidin: Forward, 5′-AAG GAA CAG GGG GTG GTG-3′ and Reverse, 5′-CCG GGA AAT TTT CTT GAA CC-3′.

### Quantification of bacterial 16S rDNA

Genomic DNA was isolated from feces containing gut microbiota using AccuPrep® Stool DNA Extraction Kit (Bioneer, Daejeon, South Korea). After the addition of each genomic DNA and 10 pmol of primers for each 16S sequence to AccuPower® 2X GreenStar™ qPCR Master Mix (Bioneer), qPCR was performed under the following conditions: 95 °C for 15 s, 55 °C for 15 s, and 72 °C for 15 s. Using 16S rDNA amplified from the qPCR, the bacterial load was assessed by copy numbers of the 16S rDNA gene per milligram of feces, and the relative numbers of bacteria in each phylum were determined by phylum-specific PCR primer pairs. The sequences of the primers used in qPCR are as follows: Universal 16S rRNA: Forward, 5′-AGA GTT TGA TCC TGG CTC-3′ and Reverse, 5′-TGC TGC CTC CCG TAG GAG T-3′; Bacteroidetes: Forward, 5′-CRA ACA GGA TTA GAT ACC CT-3′ and Reverse, 5′-GGT AAG GTT CCT CGC GTA T-3′; Firmicutes: Forward, 5′-TGA AAC TYA AAG GAA TTG ACG-3′ and Reverse, 5′-ACC ATG CAC CAC CTG TC-3′; Actinobacteria: Forward, 5′-TGT AGC GGT GGA ATG CGC-3′ and Reverse, 5′-AAT TAA GCC ACA TGC TCC GCT-3′; α-Proteobacteria: Forward, 5′-CIA GTG TAG AGG TGA AAT T-3′ and Reverse, 5′-CCC CGT CAA TTC CTT TGA GTT-3′; β-Proteobacteria: Forward, 5′-AAC GCG AAA AAC CTT ACC TAC C-3′ and Reverse, 5′-TGC CCT TTC GTA GCA ACT AGT G-3′; γ-Proteobacteria: Forward, 5′-TCG TCA GCT CGT GTY GTG A-3′ and Reverse, 5′-CGT AAG GGC CAT GAT G-3′; ε-Proteobacteria: Forward, 5′-TAG GCT TGA CAT TGA TAG AAT C-3′ and Reverse, 5′-CTT ACG AAG GCA GTC TCC TTA-3′.

### Immunohistochemistry

Tissue sections were washed twice with PBS and incubated for 10 min with 0.3% hydrogen peroxide, followed by washing three times with PBS. The tissues were blocked with PBS containing 3% bovine serum albumin for 30 min, and stained with primary antibodies for 2 h at 4 °C. After washing, the tissues were stained with a secondary antibody-conjugated horseradish peroxidase (HRP) for 1 h, washed, and added DAB substrate solution (Vector Laboratories, Newark, CA). After washing three times, the tissues were counterstained with hematoxylin for 1 min, washed; then observed at 200 × magnification under an inverted microscope. The antibodies for immunohistochemistry were as follows: anti-Axl (STJ97643; St John's Laboratory, London, UK), anti-Gas6 (sc-22759; Santa Cruz Biotechnology, Dallas, TX), anti-rabbit IgG (H + L)-HRP (31460; Thermo Fisher Scientific, Waltham, MA).

### Western blot analysis and enzyme-linked immunosorbent assay (ELISA)

The primary colon and MLN cells were lysed with cell lysis buffer (1% Triton X-100, 50 mM Tris–HCl (pH 7.4), 150 mM NaCl, and 5 mM EDTA) supplemented with the protease inhibitor cocktail (Merck, Rahway, NJ) and incubated 30 min at 4 °C. After centrifugation at 13,000×*g* for 30 min at 4 °C, the supernatant was harvested and boiled for 5 min in the presence of 5 × protein sample buffer (300 mM Tris–HCl, 20 mM EDTA, 10% SDS, 25% β-mercaptoethanol, 50% glycerol, and bromophenol blue). Western blot analysis was performed using the indicated antibodies: anti-mouse CRAMP (PA-CRPL-100; Innovagen, Lund, Sweden), anti-rabbit IgG (H + L)-HRP (31460; Thermo Fisher Scientific, Waltham, MA). Protein levels of IL-1β, IL-6 and IFN-γ were measured in the colon and MLN using ELISA kits according to the manufacturer's instruction (IL-1β; MLB00C, IL-6; M6000B, IFN-γ; MIF00, R&D Systems, Minneapolis, MN).

### Flow cytometric analysis

Cells were stained with APC-anti-mouse CD3e (553066; BD Pharmingen, San Diego, CA) and PE-anti-mouse CD4 (557308; BD Pharmingen) for 30 min at 4 °C, followed by washing twice with PBS containing 1% FBS and 0.03% sodium azide. For intracellular staining, cells were fixed and permeabilized using eBioscience™ Intracellular Fixation & Permeabilization Buffer (Thermo Fisher Scientific) followed by staining with FITC-anti-mouse IFN-γ (554411; BD Pharmingen). After washing twice with PBS containing 1% FBS and 0.03% sodium azide, the cells were analyzed by FACScalibur (Becton, Dickinson and Company, Franklin Lakes, NJ) using FlowJo software (Becton Dickinson).

### Fecal microbiota transfer (FMT)

Fresh feces from WT and Axl^−/−^ mice were suspended with PBS at a concentration of 1 mg per 10 µl and then filtered through a 70 µm nylon strainer. The fecal suspensions supplemented with 10% glycerol were stored at − 80 °C and thawed before FMT. To deplete the gut microbiota, WT and Axl^−/−^ mice were administered with antibiotics cocktail (Merck, Rahway, NJ) every other day for a week prior to FMT. For the FMT, 100 µl of fecal suspension was administered orally per mouse three times a week, followed by administration of 2.5% DSS for 5 days.

### Statistical analysis

The statistical significance values were calculated using the unpaired Student’s T-test, Mann–Whitney test, or one-way ANOVA. The data were considered statistically significant when the p-value < 0.05.

## Supplementary Information

Supplementary Figure S1.

## Data Availability

The datasets used and/or analyzed during the current study available from the corresponding author upon reasonable request.
