# Supplementary material for: Axl alleviates DSS-induced colitis by preventing dysbiosis of gut microbiota
Source: Sci Rep. 2023 Apr 1;13:5371. doi: 10.1038/s41598-023-32527-2 (PMC10067963; doi:10.1038/s41598-023-32527-2)
Supplement: Supplementary file 1 — Supplementary Figure S1. [file 41598_2023_32527_MOESM1_ESM.pptx]

## Slide 1
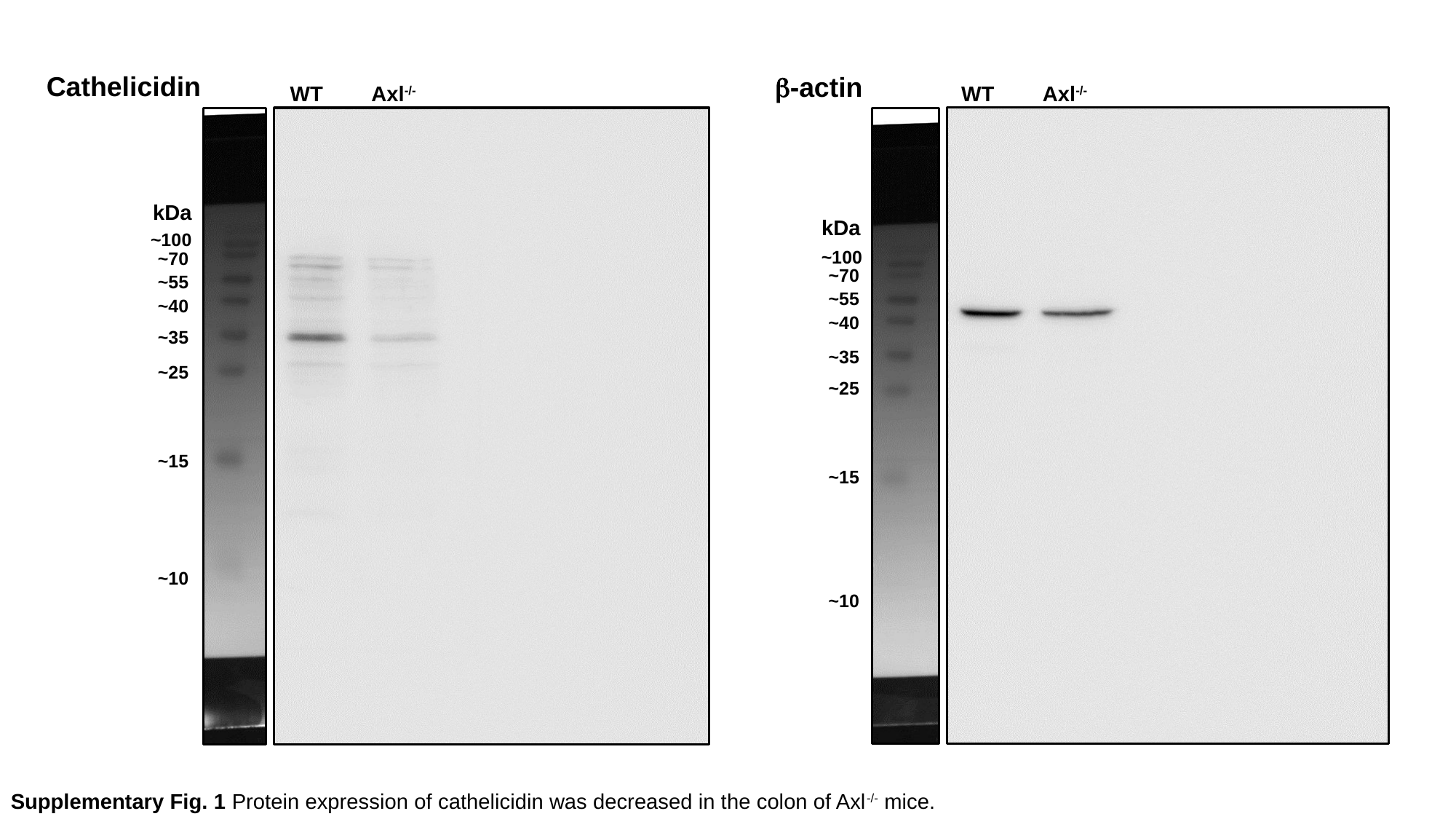

Cathelicidin
b-actin
WT
Axl-/-
WT
Axl-/-
kDa
kDa
~100
~100
~70
~70
~55
~55
~40
~40
~35
~35
~25
~25
~15
~15
~10
~10
Supplementary Fig. 1 Protein expression of cathelicidin was decreased in the colon of Axl-/- mice.
